# Contributions of the posterior cerebellum to mentalizing and social functioning: A transdiagnostic investigation

**DOI:** 10.1017/S003329172500039X

**Published:** 2025-03-03

**Authors:** Aubrey M. Moe, Scott D. Blain, Aravind Kalathil, Scott Peltier, Costanza Colombi, Katharine N. Thakkar, Cynthia Z. Burton, Ivy F. Tso

**Affiliations:** 1 Department of Psychiatry and Behavioral Health, The Ohio State University, Columbus, OH, USA; 2 Department of Psychology, The Ohio State University, Columbus, OH, USA; 3 Department of Neuroscience, The Ohio State University, Columbus, OH, USA; 4 Functional MRI Laboratory, University of Michigan, Ann Arbor, MI, USA; 5 IRCCS Stella Maris Foundation, Pisa, Italy; 6 Department of Psychology, Michigan State University, Lansing, MI, USA; 7 Department of Psychiatry, University of Michigan, Ann Arbor, MI, USA

**Keywords:** cerebellum, social cognition, psychosis, autism, social anxiety, social neuroscience, neurodevelopment

## Abstract

**Background:**

Mentalizing—our ability to make inferences about the mental states of others—is impaired across psychiatric disorders and robustly associated with functional outcomes. Mentalizing deficits have been prominently linked to aberrant activity in cortical regions considered to be part of the “social brain network” (e.g., dorsomedial prefrontal cortex, temporoparietal junction), yet emerging evidence also suggests the importance of cerebellar dysfunction. In the present study—using a transdiagnostic, clinical psychiatric sample spanning the psychosis-autism-social anxiety spectrums—we examined the role of the cerebellum in mentalizing and its unique contributions to broader social functioning.

**Methods:**

Sixty-two participants (38 with significant social dysfunction secondary to psychiatric illness and 24 nonclinical controls without social dysfunction) completed a mentalizing task during functional magnetic resonance imaging. General linear model analysis, latent variable modeling, and regression analyses were used to examine the contribution of cerebellum activation to the prediction of group status and social functioning.

**Results:**

Mentalizing activated a broad set of social cognitive brain regions, including cerebral mentalizing network (MN) nodes and posterior cerebellum. Higher posterior cerebellum activation significantly predicted clinical status (i.e., individuals with psychiatric disorders versus nonclinical controls). Finally, cerebellar activation accounted for significant variance in social functioning independent of all other cerebral MN brain regions identified in a whole-brain analysis.

**Conclusions:**

Findings add to an accumulating body of evidence establishing the unique role of the posterior cerebellum in mentalizing deficits and social dysfunction across psychiatric illnesses. Collectively, our results suggest that the posterior cerebellum should be considered – alongside established cerebral regions – as part of the mentalizing network.

## Introduction

Social dysfunction is prevalent across psychiatric conditions and leads to significant, enduring functional impairments that interfere with independent living, occupational functioning, and interpersonal relationships. Impairment in social cognition is an established key driver of social dysfunction and outcomes across psychiatric diagnoses. Social cognition – the mental processes that underlie social perception and behavior(Green et al., 2008) – is diminished in psychiatric disorders (Green et al., 2015) and contributes to impaired social and community functioning among individuals with psychiatric disorders (Fett et al., 2011) – even beyond the impact of other illness-related factors such as psychiatric symptoms and nonsocial cognition (Kalin et al., 2015; Mehl et al., 2010; Santamaría-García et al., 2020). Of all social cognitive domains, mentalizing – the ability to infer and reason about the mental states of others (Biedermann et al., 2012) – is both a robustly impaired aspect of social cognition across people who experience social dysfunction secondary to psychiatric disorders (i.e., psychotic disorders [Savla et al., 2013], social anxiety [Sloover et al., 2022], and autism spectrum disorders [Chung et al., 2014]) and is consistently associated with and predictive of functional (Fett et al., 2011; Hoogenhout & Malcolm-Smith, 2017; Roncone et al., 2002) and social outcomes.(Mike et al., 2019). Thus, understanding the mechanisms underlying mentalizing ability may provide critical insight on how to more effectively intervene to address social dysfunction among people living with psychiatric disorders.

Mentalizing is associated with a network of cerebral brain regions including the medial prefrontal cortex (mPFC), precuneus, temporoparietal junction (TPJ), and superior temporal sulcus (STS) that are consistently activated during tasks that require making inferences about the mental states of other people (Arioli et al., 2021; Frith & Frith, 2006; Van Overwalle, 2009). Though the vast majority of neuroimaging studies investigating mentalizing have been limited to cerebral brain regions, an accumulating body of evidence suggests that the cerebellum plays an important and previously unrecognized role in social cognitive processes. The cerebellum – a major structure of the hindbrain that sits beneath the cerebrum – has historically been recognized as contributing to motor behavior (Buckner, 2013; Middleton & Strick, 1998). Alongside more recent studies noting important cerebellar contributions to cognition and affect (Glickstein, 2007; Mariën et al., 2014) a specific region of the posterior cerebellum (Crus II) has been identified as playing a key role in mentalizing among healthy, nonclinical adult participants (Heleven et al., 2019; Van Overwalle et al., 2015; Van Overwalle, Ma, et al., 2020). These observations are consistent with evidence that patients with degenerative cerebellar disorders demonstrate gray matter reduction in Crus II and impairments in mentalizing function (Clausi et al., 2019).

A rapidly developing view in the field is that Crus II of the cerebellum assists with learning and understanding social action sequences (Van Overwalle, Manto, et al., 2020) and thus facilitates mentalizing by creating predictions about the social behaviors and intentions of others (Haihambo et al., 2022; Van Overwalle, 2009) More specifically, the posterior cerebellum may be a *forward controller* that checks information extracted from the cerebrum against internal social sequencing models to subsequently improve predictions about the social behavior or information being processed (Haihambo et al., 2024; Heleven et al., 2019; Van Overwalle et al., 2015, 2021). This hypothesized role of the posterior cerebellum has particular relevance for understanding the neural underpinnings of the mentalizing impairments observed among individuals living with psychiatric disorders, which often involve ignoring/misinterpreting social cues and an impaired ability to implicitly learn and automatize predictions about social-behavioral patterns (i.e., abnormal mentalizing; Van Overwalle et al., 2021). In summary, the posterior cerebellum appears to interface with the cerebrum to support social cognitive function. However, its contributions to mentalizing and social deficits among individuals with psychiatric disorders remains relatively unexplored.

Thus, our primary objective in the present study was to examine posterior cerebellar contributions to mentalizing in psychopathology. We investigated this in a young (ages 14-30), transdiagnostic sample of individuals with significant social dysfunction secondary to a psychiatric illness. The use of a transdiagnostic sample enriched for social dysfunction permits the examination of shared and unique mechanisms of social dysfunction across psychiatric disorders. This age group, where most psychiatric disorders emerge and escalate, allows us to better determine the presence and extent of cerebellar abnormalities near illness onset and mitigate potential confounds of longer-standing illness. We hypothesized that Crus II of the cerebellum – alongside other known cerebral mentalizing network (MN) regions – would be activated during mentalizing, and further that brain activation in this region would significantly predict participant group status (i.e., differentiate individuals with psychiatric disorders from healthy control participants) beyond the contribution of other cerebral mentalizing regions. Finally, we hypothesized that activation in the posterior cerebellum during mentalizing would significantly account for individual differences in social functioning, over and above all other cerebral regions implicated in mentalizing.

## Method

*Participants.* Data from participants with significant social dysfunction secondary to psychiatric illness (n = 38) and nonclinical controls (n = 24) were drawn from a larger neuroimaging study examining altered social cognitive processing as a biobehavioral marker of social dysfunction spanning psychiatric diagnoses (Tso et al., 2020; National Institute of Mental Health, R01MH122491). Eligibility criteria for all participants were (1) age 14 to 30; (2) ability and willingness to give informed consent; (3) vision equal to or better than 20/30 on the Snellen visual acuity test, with correction if necessary; (4) no significant neurological abnormalities, such as seizure disorder, mass lesions, etc.; (5) no known Mendelian disorder; and (6) no active substance abuse in the past 30 days (as determined by the Mini International Neuropsychiatric Interview (MINI; Lecrubier et al., 1997); (7) IQ ≥ 80 (assessed via Wechsler Abbreviated Scale of Intelligence – Second Edition; McCrimmon & Smith, 2013).

Participants in the psychiatric illness group had a DSM-5 psychiatric diagnosis confirmed with the MINI and significant social difficulties (defined as either Global Functioning – Social [GF-Social; Piskulic et al., 2011] score ≤ 6 or a score of ≤ 69 on the Mental Illness Research Education and Clinical Center (MIRECC) Global Assessment of Functioning [GAF; Niv et al., 2007]). This group was further enriched on the psychosis, autism, and social anxiety spectrums (i.e., 92% of the psychiatric participants met clinical criteria for one or more of these disorders) to ensure that social dysfunction is not merely due to low motivation (such as that commonly occurring in depression).

Individuals in the nonclinical control group met the additional eligibility criteria of (1) no history of past or current DSM-5 diagnosis; (2) no social impairment (GF-Social score of ≥7 and ≥70 on MIRECC GAF[Niv et al., 2007]; and (3) not currently taking any psychotropic medications.

All study participants provided written informed consent prior to their study participation. This study was approved by the University of Michigan Institutional Review Board. The authors assert that all procedures contributing to this work comply with the ethical standards of the relevant national and institutional committees on human experimentation and with the Helsinki Declaration of 1975, as revised in 2008.

**Figure 1.**
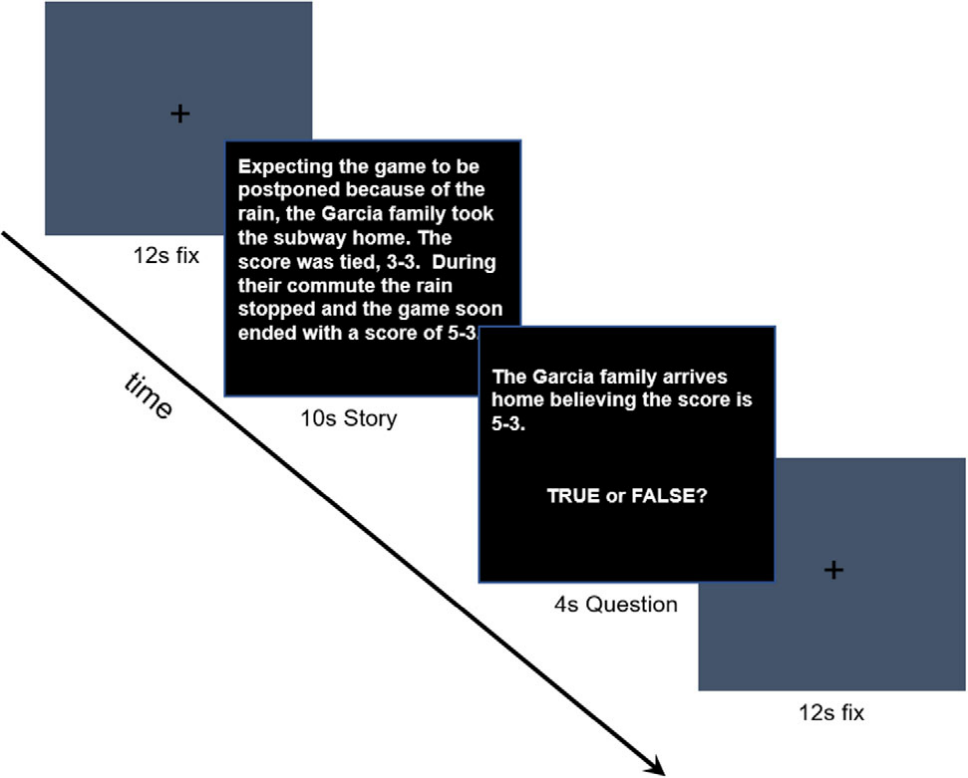
False-belief task. During the task, short stories are presented that require representation of false content, either by making a prediction about a person’s future behavior or thinking based on belief representation (belief condition) or by reasoning about false or outdated physical states in photographs or maps (photo condition).

*fMRI Task, Acquisition, and Preprocessing.* Participants completed an in-scanner task of social mentalizing: the false-belief task (FBT; Figure 1). FBT is a well-validated task that taps high-level mentalizing, that is, attribution of beliefs (Dodell-Feder et al., 2011). Participants are presented with a short (2-3 sentence) story, followed by a statement about that story to which they must respond ‘true’ or ‘false’ by pressing a button. There are two conditions to the task: BELIEF and PHOTO. Under the BELIEF condition, participants are presented with statements about a person and when responding to the subsequent statement, they must take on the perspective of the person in the story to accurately answer the TRUE/FALSE statement. Under the PHOTO condition, participants are also presented with brief stories, but this time, they are presented with a statement describing a drawing or photograph and must respond whether the statement is TRUE/FALSE. Both sets of stories require participants to read short stories and represent false content; the critical difference is in the type of false content represented (i.e., a belief versus a photograph/map). Stories were followed by a true/false question that referred either to the situation in reality or to the false representation. During the FBT, participants complete two runs, each consisting of 5 stories from each condition. Stories are presented for 10s, followed by a true/false question for 4s, and then 12s of center-cross fixation. Accuracy on the FBT was calculated by the number of correct TRUE/FALSE responses across all trials of the task, with separate calculations for the BELIEF and PHOTO conditions.

MRI scanning occurred on a 3.0 T GE MR Discovery scanner with a multi-band EPI sequence for functional images (gradient echo, TR=800 ms, TE=30 ms, FA=52 degrees, FOV=21.6 cm, 60 slice, 2.4-mm thick/0-mm skip, hyperband slice factor = 6, equivalent to 90 × 90 voxel grid). Results included in this manuscript come from preprocessing performed using *fMRIPrep* (Esteban et al., 2019; Gorgolewski et al., 2011), which is based on *Nipype* 1.5.1 (Gorgolewski et al., 2011). Complete details on anatomical and functional data preprocessing appear in Supplementary Materials.

*Social Functioning.* A latent variable of social functioning was computed using the “fa” function from the “psych” package in R(Revelle, 2015) across data available from all participants in the larger study dataset (n = 135). Factor scores for social functioning were computed using a single-factor exploratory factor analysis with minimum residual factoring, followed by the regression method of factor score estimation. Indicator variables for social functioning included one self-report and two interviewer-rated measures. Ratings for each of the interviewer-rated measures were supervised and reviewed by a licensed clinical psychologist on a weekly basis.

Work and Social Adjustment Scale (WSAS; Zahra et al., 2014). The WSAS is a 4-item, self-report measure which assesses an individual’s perspective of their current level of functional impairment attributable specifically to psychiatric symptoms. Each item on the WSAS is rated on a scale from 1 to 8, with higher scores indicating more severe dysfunction. In the present study, we utilized the sum of the two social items of the WSAS.

MIRECC Social Functioning Scale (Niv et al., 2007). The MIRECC Global Assessment of Functioning Scale is an interviewer-rated measure that includes 3 separate subscales assessing an individual’s occupational functioning, social functioning, and symptom severity over the previous month. Given our interest in assessing social dysfunction, we utilized the social functioning subscale in the present study. Scores on this measure range from 1 to 100, with higher scores reflecting better social functioning.

GF-Social Scale (GF-S; Piskulic et al., 2011). The GF-S is an interviewer-rated assessment of interpersonal functioning at multiple timepoints in the preceding year (i.e., current, best in the past year, and worst in the past year. We utilized the worst in the past year score in the present study to both convey the range of each individual’s social dysfunction and complement the MIRECC Social Functioning score. Scores on this measure range from 1 to 10, with higher scores reflecting better social functioning.

## Analyses

Brain activity associated with mentalizing was identified using general linear model (GLM) analysis of the fMRI time series data. The anatomically normalized time series was regressed on two regressors of interest (represented using boxcar regressors timed to blocks for the BELIEF and PHOTO conditions), nuisance regressors (motion correction and runs), and convolved with a canonical hemodynamic response function for each participant. For the second-level models, voxel clusters that were commonly activated across all participants as well as those differentially activated between those with and without significant social dysfunction were examined. Models identified voxels with significantly different activation between the BELIEF vs PHOTO conditions at a cluster-defining threshold of p < 0.001 and an FWE correction of p < 0.05. Second-level clusters were labeled to the Automated Anatomical Labeling 3 Atlas using the Python package ‘atlasreader’ (Notter et al., 2019). After examining significant clusters for the BELIEF-PHOTO GLM contrast across all participants in the sample, beta estimates within an 8-mm radius sphere from the peaks of significant clusters were extracted and used to statistically predict behavioral variables of interest.

To examine the unique contribution of cerebellum activation in predicting group status, activation estimates for each region were included as predictors in a logistic regression model statistically predicting group membership (i.e., individuals with psychiatric diagnoses or nonclinical control participants). The dependent variable was a dummy variable with nonclinical controls coded as 0 and patients coded as 1. Follow-up sensitivity analyses were conducted that also included sex and age as covariates.

Next, to test our hypothesis that cerebellar activation would predict unique variance in social functioning, we conducted a hierarchical regression analysis. In the first block of this regression model, our reduced model included extracted activation (beta estimate of the BELIEF-PHOTO contrast) from each of the cerebral brain regions identified in our whole-brain analysis as predictors, with the latent factor of social functioning entered as the dependent variable. In the second block, extracted cerebellar activation was added to assess whether the full model accounted for additional variance. Consistent with our group regression analyses, sex and age were included as covariates in subsequent sensitivity analyses.

## Results

### Participant demographics

Participants self-identified as Asian (23%), Black (5%), multiracial (1%), or White (71%). Additional participant demographics are as follows: *M* age = 23.606 (+ 3.804; range = 16 to 30), males = 15 (23%) and females = 51 (77%). Full demographics for each group and clinical characteristics appear in Table 1.

**Table 1.** Group demographics and clinical characteristics

	Psychiatric patients	Nonclinical controls	Group difference statistics
N	38	24	
Mean age (SD)	23.95 (4.09)	23.08 (3.32)	t(60) = .869, p = .388
Biological sex (% Female)	84%	67%	*X* ^2^ = 2.590, df = 1, p = .108
Race (%)			*X* ^2^ = 5.409, df = 3, p = .144
White	30 (79%)	14 (58%)	
Black	2 (5%)	1 (4%)	
Asian	6 (16%)	9 (38%)	
Primary diagnosis			
Autism spectrum disorder	14 (37%)		
Psychotic disorder	4 (8%)		
Social anxiety disorder	18 (50%)		
Other clinical^a^	2 (8%)		
Any major depressive disorder diagnosis	5 (13%)		

a Other clinical participants did not meet criteria for the primary study diagnoses (i.e., autism, psychosis, or social anxiety) but did meet criteria for other psychiatric diagnoses (Major Depressive Disorder or Generalized Anxiety Disorder).

### Behavioral results

All indicators loaded strongly and significantly onto the social functioning factor. Factor loadings were highest for GF-Social (*λ* = .96), followed by MIRECC Social Functioning (*λ* = .95) and then WSAS (*λ* = -.90). Higher scores on the factor represented higher levels of social functioning.

Performance on the belief condition of the FBT was significantly associated with the social functioning factor score (*β* = .302, *p* = .022). This association held when sex and age were included as covariates (*β* = .287, *p* = .033). Given the presence of two potential outliers, analyses were repeated using a Spearman partial correlation; the effect remained significant (*ρ_partial_* = .230, *p* = .035).

### fMRI results

**Figure 2.**
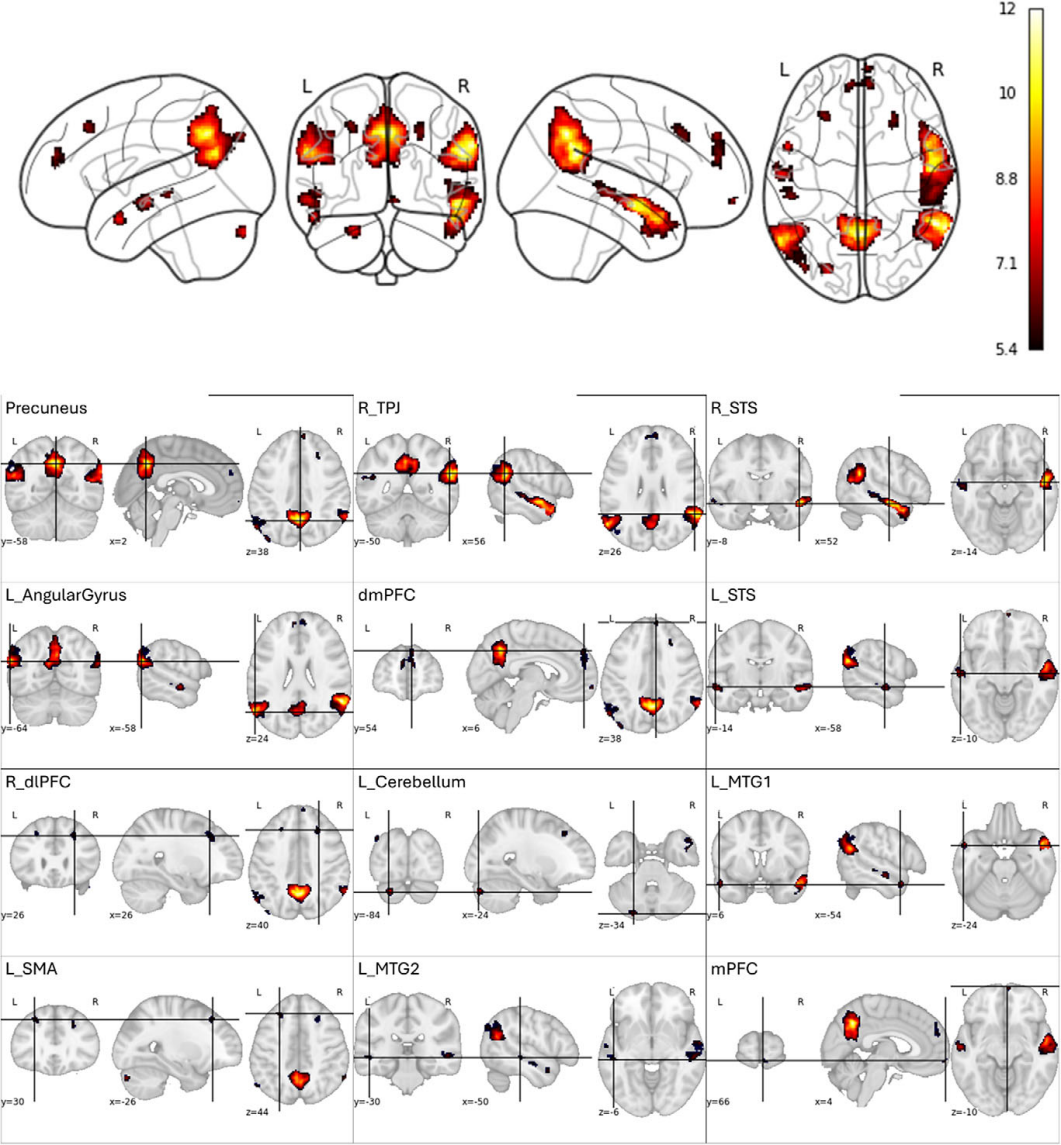
Brain regions significantly activated during mentalizing. *T*-map (*p* < 0.001) rendered on a canonical brain image highlighting regions that were more active during the Belief condition than the Photo condition (of the false-belief task) and a bar graph of extracted BOLD response. This figure shows general linear model results for all participants. When present, R = right and L = left. TPJ = temporoparietal junction. STS = superior temporal sulcus. MTG = middle temporal gyrus. mPFC = medial prefrontal cortex. dmPFC = dorsomedial prefrontal cortex. SMA = supplementary motor area. dlPFC = dorsolateral prefrontal cortex.

Mentalizing (identified using the BELIEF>PHOTO GLM contrast) was associated with greater activation in a variety of brain regions canonically associated with social cognition (e.g., TPJ, precuneus, STS) and in a voxel-cluster within Crus II of the posterior cerebellum. Results are visualized in Figure 2 and fully described in Table S1.

Results of logistic regression analyses statistically predicting group status using all brain activation estimates identified in our whole-brain analyses as simultaneous predictors are presented in Table 2. Activation of the posterior cerebellum was predictive of group status (*β* = 2.466, p = .016), such that those with higher cerebellum activation are more likely to be psychiatric patients than the nonclinical controls. Posterior cerebellum activation remained a significant predictor even when age and sex were included as covariates (Table S2).

**Table 2.** Logistic regression model of brain activation predictors and group status^a^

Brain region and AAL^b^ coordinates	*Β^c^*	*p*
R_TPJ [56, –50, 26]	–0.842	.338
Precuneus [2, –58, 38]	–1.120	.304
R_STS [52, –8, –14]	1.396	.194
L_AngularGyrus [–58, –64, 24]	0. 831	.442
L_MTG1 [–54, 6, –24]	1.902	.036*
L_Cerebellum [–24, –84, –34]	2.466	.016*
L_STS [–58, –14, –10]	–2.978	.019*
mPFC [4, 66, –10]	0.643	.468
dmPFC [6, 54, 38]	–0.965	.363
L_SMA [–26, 30, 44]	1.578	.153
L_MTG2 [–50, –30, –6]	–0.078	.919
R_dlPFC [26, 26, 40]	–2.126	.032*

* p < .05.

a Group status indicates prediction of psychiatric patient versus nonclinical control.

b Automatic Anatomical Labeling.

c Standardized regression coefficients; positive coefficients convey activations greater in patients relative to controls. Results controlling for sex and age are presented in the supplement (Table S1). When present, R = right and L = left. TPJ = temporoparietal junction. STS = superior temporal sulcus. mPFC = medial prefrontal cortex. MTG = middle temporal gyrus. dmPFC = dorsomedial prefrontal cortex. SMA = supplementary motor area. dlPFC = dorsolateral prefrontal cortex.

Results of hierarchical regression analyses examining the unique prediction of social functioning by cerebellum activation appear in Table 3. After accounting for the variance explained by all cerebral nodes identified in the whole-brain analysis in the reduced model (16.9%), the addition of posterior cerebellum activation in the full model explained significantly more variance in social functioning (ΔR^2^= 6.7%, p = .045.). More specifically, poorer social functioning was significantly predicted by *higher* activation in Crus II of the left cerebellum (β = -.400, p = .046). Though the general pattern of results remained when age and sex were included as covariates, the unique contribution of the posterior cerebellum (ΔR^2^ = 6.0%) no longer reached statistical significance, p = .057 (Table S3).

**Table 3.** Hierarchical linear regression of regional activation as predictors of social functioning

Model		Model statistics			Predictor statistics	
		R^2^(ΔR^2^)	F	*p*	β	*p*
*Model 1*		0.169 (0.169)	0.967	0.489		
R_TPJ					0.000	0.999
Precuneus					0.075	0.736
R_STS					–0.164	0.463
L_Angular Gyrus					–0.015	0.951
L_MTG					–0.166	0.348
L_STS					0.447	0.045*
mPFC					–0.179	0.364
dmPFC					–0.079	0.717
L_SMA					–0.159	0.454
L_MTG2					–0.051	0.762
R_dlPFC					0.246	0.186
*Model 2*		0.236 (0.067)	4.219	0.045*		
R_TPJ					0.069	0.710
Precuneus					0.228	0.322
R_STS					–0.219	0.316
L_Angular Gyrus					–0.040	0.868
L_MTG					–0.257	0.149
L_STS					0.586	0.011*
mPFC					–0.171	0.371
dmPFC					0.063	0.778
L_SMA					–0.337	0.136
L_MTG2					–0.054	0.741
R_dlPFC					0.353	0.063
L_Cerebellum					–0.397	0.046*

*Note*: *p < .05. When present, R = right and L = left. TPJ = temporoparietal junction. STS = superior temporal sulcus. mPFC = medial prefrontal cortex. MTG = middle temporal gyrus. dmPFC = dorsomedial prefrontal cortex. SMA = supplementary motor area. dlPFC = dorsolateral prefrontal cortex.

## Discussion

In the present study, activation was observed in a broad set of social cognitive brain regions – including key cerebral nodes of the MN (i.e., rTPJ, precuneus, STS, dmPFC) and the posterior cerebellum (Crus II) during social mentalizing. Activation in the posterior cerebellum during mentalizing significantly differentiated individuals with psychiatric disorders and nonclinical control participants when all brain regions were included in the model. Finally, activation in the posterior cerebellum predicted unique variance in social functioning independent of activation in all other cerebral regions in our transdiagnostic sample of individuals with varying levels of social functioning.

These results add to an accumulating body of evidence that the posterior cerebellum plays an important role in mentalizing. First, our data suggest the Crus II of the cerebellum is activated alongside key cerebral nodes of the MN (i.e., TPJ, precuneus, STS, and dmPFC) during social mentalizing. Though activation of these regions during the mentalizing task used in this study is expected based on previous neuroimaging studies, observing this same pattern in our own data serves as a confirmation that FBT engaged the MN in our transdiagnostic sample of individuals with and without psychiatric disorders. Although several previous studies investigated MN activation during FBT in serious mental illness, they often focused on a priori region of interest (ROI) approaches limited to the cerebrum only (Dodell-Feder, DeLisi, et al., 2014; Hegde et al., 2021). Our approach extends previous research by utilizing a rigorous, whole-brain analysis that permitted detection of important cerebellar contributions to mentalizing in addition to traditional social regions in the cerebrum. In a recent neuroimaging study utilizing the FBT (Dodell-Feder, Tully, et al., 2014), both individuals with schizophrenia and nonclinical controls showed cerebellar activation in Crus II during mentalizing – but these activations were not significantly different between groups nor were possible associations between cerebellar activation and social functioning reported. The findings of the current study thus highlight both the relevance of the posterior cerebellum in mentalizing as well as the need for future studies to more deliberately examine and report the associations of cerebellar function – or lack thereof – with other measures of key functional and clinical outcomes among individuals with psychiatric diagnoses.

We found abnormally increased activation of the posterior cerebellum among individuals with psychiatric illness, which in turn explained *unique variance in social functioning* when all cerebral regions were also considered in the model. These findings warrant consideration of what role the posterior cerebellum plays or supports during mentalizing. Van Overwalle and colleagues, in a series of rigorous studies and meta-analyses with nonclinical populations, have noted that Crus II of the cerebellum is selectively activated during mentalizing to not only predict social sequences to facilitate social behavior and anticipate outcomes of upcoming social interactions but also to engage in error signaling to cerebral regions of the brain when these predictions are not upheld or otherwise do not align with external consequences (Heleven et al., 2019, 2021; Van Overwalle et al., 2014; Van Overwalle, Ma, et al., 2020; Van Overwalle, Manto, et al., 2019; Van Overwalle, Van de Steen, et al., 2019; Van Overwalle & Mariën, 2016). They outline that this important error signal model would permit real-time adjustment of internal and external behaviors to better adapt to the situation or social demands – which is key to social survival, interaction, and reward (Van Overwalle & Heleven, 2023). If the posterior cerebellum does behave as an error signaler to cerebral regions involved in social cognitive processing, increased activation of this region in our group of individuals with psychiatric illness may reflect *increased error activity or error processing* when engaging in mentalizing. It is possible that dysfunction in predicting, monitoring social interactions, and/or re-aligning social behaviors when engaging in mentalizing contributes to increased engagement of the posterior cerebellum, which is tasked with identifying and rectifying erroneous social sequence predictions in real time. This interpretation is both consistent with findings from a previous study supporting the role of the posterior cerebellum in social sequencing (Heleven et al., 2021) and further bolstered by our observation that increased cerebellar activation is both predictive of patient-versus-control status and related to poorer social functioning across participants. Thus, the present study provides preliminary evidence that the posterior cerebellum may be considered as a relevant node of the established cerebral MN. This possibility can be further probed in studies which investigate the dynamic interactions of the posterior cerebellum and cerebral MN brain regions while engaged in mentalizing.

Critically, our results suggest that individuals with psychiatric disorders have significantly *greater* activation of the posterior cerebellum during mentalizing that differentiates them from nonclinical control participants. We should note that our finding of increased activation of the posterior cerebellum during mentalizing in patients with socially impairing psychiatric disorders does not align with the majority of studies examining cerebellar activation among individuals with serious mental illness, which have shown reduced recruitment of this brain region across a variety of different tasks (e.g., perceptual, motor, cognitive, and emotional; Lungu et al., 2013). Future research should investigate the influence of specific types of social cognitive tasks (e.g., a story-reading task like the one utilized in this study compared to tasks that are more interactive or feature interpersonal/human stimuli) or clinical presentation (e.g., psychiatric patients with prominent social dysfunction compared to patients with better functioning) to further clarify our pattern of results.

It is important to acknowledge the results that were not consistent with our predictions. Notably, though posterior cerebellum activation predicted unique variance in social functioning, these results became slightly weaker and did not reach statistical significance after controlling for age and sex. Studies in animal (Nguon et al., 2005) and nonclinical human populations (Fan et al., 2010; Işıklar et al., 2023; Tiemeier et al., 2010) have noted cerebellar sexual dimorphisms, with biological females and males demonstrating discrepant developmental trajectories with regard to cerebellar structure and function during the years spanning childhood and adolescence. To fully understand the role of the cerebellum in social functioning, future studies with larger sample size should clarify these results by examining possible differences in cerebellar activation as a function of developmental stage or biological sex.

This study has several notable strengths. First, our utilization of a transdiagnostic group of individuals with psychiatric disorders selected specifically for social dysfunction (rather than those with specific psychiatric diagnoses) permits examination of common mechanisms of the neural underpinnings of social dysfunction across mental disorders. Furthermore, the presence of both adolescent and young adult participants in this dataset provides an opportunity to understand mentalizing during the phases of life when most psychiatric disorders emerge and escalate while also mitigating potential confounds of longer-standing illness. Next, we utilized a well-validated task to probe mentalizing, thus maximizing our ability to understand our pattern of results relative to other studies with different clinical populations. Finally, our use of a latent factor of social functioning variable integrating multiple measures from multiple sources permits an understanding of how neural activation relates to a broader and more reliable index of social capacity.

Our study also has several limitations to consider. First, the cross-sectional nature of this study does not permit inferences about how cerebellar function may vary over time or course of illness within an individual. Though our use of a latent social functioning variable permitted an evaluation of neural contributions to broad social outcomes, future research may clarify whether more specific aspects of social functioning may be associated with cerebellar function. Next, though our use of a transdiagnostic sample likely improves external validity of our results due to the high rates of psychiatric comorbidity among individuals spanning the psychosis-autism-anxiety spectrums (Koyuncu et al., 2019; Mannion & Leader, 2013; Wilson et al., 2020), it also limits our ability to make inferences about the impact of specific psychiatric diagnoses. Additionally, a small subsample of individuals in the psychiatric participant group also met criteria for a primary (n=1) or comorbid (n=4) major depressive disorder. Though the majority of participants thus did not have clinical depression it is unlikely that our pattern of results reflects social dysfunction attributable to a mood process, this possibility should be considered and more thoroughly assessed in future studies. Though our use of a nonclinical control group in the present study permitted comparison of these individuals to participants in the psychiatric group, future studies also including an additional psychiatric control group without significant social dysfunction would permit more specific inferences about the contribution of diagnostic status to cerebellar function. Finally, we utilized a sample of adolescents and young adults in this study, and thus, it is unclear how cerebellar function may differ in different developmental phases of life. Future studies which employ longitudinal designs and/or span wide age ranges may further delineate the impact of development/aging and provide important insights with regard to the role of the cerebellum in mentalizing across the lifespan.

In summary, the current study provides a preliminary characterization of cerebellar contributions to mentalizing and social dysfunction. Alongside emerging research establishing structural abnormalities in Crus II of the posterior cerebellum and their associations with behavioral social cognitive performance among individuals with various forms of psychopathology (Clausi et al., 2019; Olivito et al., 2023), our data have important implications for future research. First, future studies should investigate the dynamic interactions of the cerebellum with known cerebral regions implicated in social cognition. Critically, this type of research has potential translational applications to intervention for social dysfunction in mental illness. Given the proximity of the cerebellum to the scalp relative to cerebral regions of the MN located deeper within the brain (e.g., mPFC, precuneus), targeting the posterior cerebellum is both more accessible for neuromodulation and could have downstream effects on cerebral MN activity otherwise not accessible via neuromodulation. Additionally, the current results may have implications for psychosocial interventions. More specifically, the effectiveness of therapies intended to improve mentalizing may be optimized if they also focus on improving the ability to identify and predict social sequences and understand the related perspectives of social partners. Narrative sequencing therapy, for example, has recently been shown to improve mentalizing among adults with autism spectrum disorders (Bylemans et al., 2022). Given both the probable role of the cerebellum in social sequencing and the relevance of the cerebellum in predicting social dysfunction in our transdiagnostic sample, similar psychotherapies may be helpful for a range of psychiatric problems impacting social dysfunction.

## Supporting information

Moe et al. supplementary material Moe et al. supplementary material

## Supplementary material

To view supplementary material for this article, please visit http://doi.org/10.1017/S003329172500039X.
