# Supplementary material for: Contributions of the posterior cerebellum to mentalizing and social functioning: A transdiagnostic investigation
Source: Psychol Med. 2025 Mar 3;55:e67. doi: 10.1017/S003329172500039X (PMC12080638; doi:10.1017/S003329172500039X)
Supplement: Moe et al. supplementary material [file S003329172500039Xsup001.docx]

SUPPLEMENTARY MATERIALS

Contributions of the Posterior Cerebellum to Mentalizing and Social Functioning: A Transdiagnostic Investigation

Moe et al.

**Anatomical data preprocessing**

A total of 1 T1-weighted (T1w) images were found within the input BIDS dataset. The T1-weighted (T1w) image was corrected for intensity non-uniformity (INU) with N4BiasFieldCorrection,^1^ distributed with ANTs 2.3.3^2^ and used as T1w-reference throughout the workflow. The T1w-reference was then skull-stripped with a *Nipype* implementation of the antsBrainExtraction.sh workflow (from ANTs), using OASIS30ANTs as target template. Brain tissue segmentation of cerebrospinal fluid (CSF), white-matter (WM) and gray-matter (GM) was performed on the brain-extracted T1w using fast (FSL 5.0.9).^3^ Brain surfaces were reconstructed using recon-all (FreeSurfer 6.0.1)^4^, and the brain mask estimated previously was refined with a custom variation of the method to reconcile ANTs-derived and FreeSurfer-derived segmentations of the cortical gray-matter of Mindboggle (RRID:SCR_002438)^5^. Volume-based spatial normalization to two standard spaces (MNI152NLin6Asym, MNI152NLin2009cAsym) was performed through nonlinear registration with antsRegistration (ANTs 2.3.3), using brain-extracted versions of both T1w reference and the T1w template. The following templates were selected for spatial normalization: *FSL’s MNI ICBM 152 non-linear 6th Generation Asymmetric Average Brain Stereotaxic Registration Model,*^6^ RRID:SCR_002823; TemplateFlow ID: MNI152NLin6Asym], *ICBM 152 Nonlinear Asymmetrical template version 2009c* [RRID:SCR_008796; TemplateFlow ID: MNI152NLin2009cAsym].^7^

**Functional data preprocessing**

For each of the 6 BOLD runs per subject (across all tasks and sessions), the following preprocessing was performed. First, a reference volume and its skull-stripped version were generated using a custom methodology of *fMRIPrep*. A deformation field to correct for susceptibility distortions was estimated based on *fMRIPrep*’s *fieldmap-less* approach. The deformation field is that resulting from co-registering the BOLD reference to the same-subject T1w-reference with its intensity inverted.^8,9^ Registration is performed with antsRegistration (ANTs 2.3.3), and the process regularized by constraining deformation to be nonzero only along the phase-encoding direction and modulated with an average fieldmap template.^10^ Based on the estimated susceptibility distortion, a corrected EPI (echo-planar imaging) reference was calculated for a more accurate co-registration with the anatomical reference. The BOLD reference was then co-registered to the T1w reference using bbregister (FreeSurfer) which implements boundary-based registration.^11^ Co-registration was configured with six degrees of freedom. Head-motion parameters with respect to the BOLD reference (transformation matrices, and six corresponding rotation and translation parameters) are estimated before any spatiotemporal filtering using mcflirt (FSL 5.0.9).^12^ The BOLD time-series were resampled onto the following surfaces (FreeSurfer reconstruction nomenclature): *fsaverage*. The BOLD time-series (including slice-timing correction when applied) were resampled onto their original, native space by applying a single, composite transform to correct for head-motion and susceptibility distortions. These resampled BOLD time-series will be referred to as *preprocessed BOLD in original space*, or just *preprocessed BOLD*. The BOLD time-series were resampled into standard space, generating a *preprocessed BOLD run in MNI152NLin6Asym space*. First, a reference volume and its skull-stripped version were generated using a custom methodology of *fMRIPrep*. *Grayordinates* files^13^ containing 91k samples were also generated using the highest-resolution fsaverage as intermediate standardized surface space. Automatic removal of motion artifacts using independent component analysis (ICA-AROMA)^14^ was performed on the *preprocessed BOLD on MNI space* time-series after removal of non-steady state volumes and spatial smoothing with an isotropic, Gaussian kernel of 6mm FWHM (full-width half-maximum). Corresponding “non-aggresively” denoised runs were produced after such smoothing. Additionally, the “aggressive” noise-regressors were collected and placed in the corresponding confounds file. Several confounding time-series were calculated based on the *preprocessed BOLD*: framewise displacement (FD), DVARS and three region-wise global signals. FD was computed using two formulations following Power (absolute sum of relative motions, Power et al.^15^ and Jenkinson (relative root mean square displacement between affines, Jenkinson et al.^12^ FD and DVARS are calculated for each functional run, both using their implementations in *Nipype* (following the definitions by Power et al.^15^). The three global signals are extracted within the CSF, the WM, and the whole-brain masks. Additionally, a set of physiological regressors were extracted to allow for component-based noise correction (*CompCor*)^16^. Principal components are estimated after high-pass filtering the *preprocessed BOLD* time-series (using a discrete cosine filter with 128s cut-off) for the two *CompCor* variants: temporal (tCompCor) and anatomical (aCompCor). tCompCor components are then calculated from the top 2% variable voxels within the brain mask. For aCompCor, three probabilistic masks (CSF, WM and combined CSF+WM) are generated in anatomical space. The implementation differs from that of Behzadi et al. in that instead of eroding the masks by 2 pixels on BOLD space, the aCompCor masks are subtracted a mask of pixels that likely contain a volume fraction of GM. This mask is obtained by dilating a GM mask extracted from the FreeSurfer’s *aseg* segmentation, and it ensures components are not extracted from voxels containing a minimal fraction of GM. Finally, these masks are resampled into BOLD space and binarized by thresholding at 0.99 (as in the original implementation). Components are also calculated separately within the WM and CSF masks. For each CompCor decomposition, the *k* components with the largest singular values are retained, such that the retained components’ time series are sufficient to explain 50 percent of variance across the nuisance mask (CSF, WM, combined, or temporal). The remaining components are dropped from consideration. The head-motion estimates calculated in the correction step were also placed within the corresponding confounds file. The confound time series derived from head motion estimates and global signals were expanded with the inclusion of temporal derivatives and quadratic terms for each.^17^ Frames that exceeded a threshold of 0.5 mm FD or 1.5 standardised DVARS were annotated as motion outliers. All resamplings can be performed with *a single interpolation step* by composing all the pertinent transformations (i.e. head-motion transform matrices, susceptibility distortion correction when available, and co-registrations to anatomical and output spaces). Gridded (volumetric) resamplings were performed using antsApplyTransforms (ANTs), configured with Lanczos interpolation to minimize the smoothing effects of other kernels.^18^ Non-gridded (surface) resamplings were performed using mri_vol2surf (FreeSurfer).

Many internal operations of *fMRIPrep* use *Nilearn* 0.6.2 (RRID:SCR_001362)^19^, mostly within the functional processing workflow. For more details of the pipeline, see [the section corresponding to workflows in *fMRIPrep*’s documentation](https://fmriprep.readthedocs.io/en/latest/workflows.html).

References:

1. Tustison NJ, Avants BB, Cook PA, et al. N4ITK: Improved N3 bias correction. *IEEE Trans Med Imaging*. 2010;29(6). doi:10.1109/TMI.2010.2046908

2. Avants BB, Epstein CL, Grossman M, Gee JC. Symmetric diffeomorphic image registration with cross-correlation: Evaluating automated labeling of elderly and neurodegenerative brain. *Med Image Anal*. 2008;12(1). doi:10.1016/j.media.2007.06.004

3. Zhang Y, Brady M, Smith S. Segmentation of brain MR images through a hidden Markov random field model and the expectation-maximization algorithm. *IEEE Trans Med Imaging*. 2001;20(1). doi:10.1109/42.906424

4. Dale AM, Fischl B, Sereno MI. Cortical surface-based analysis: I. Segmentation and surface reconstruction. *Neuroimage*. 1999;9(2). doi:10.1006/nimg.1998.0395

5. Klein A, Ghosh SS, Bao FS, et al. Mindboggling morphometry of human brains. *PLoS Comput Biol*. 2017;13(2). doi:10.1371/journal.pcbi.1005350

6. Evans AC, Janke AL, Collins DL, Baillet S. Brain templates and atlases. *Neuroimage*. 2012;62(2). doi:10.1016/j.neuroimage.2012.01.024

7. Fonov V, Evans A, McKinstry R, Almli C, Collins D. Unbiased nonlinear average age-appropriate brain templates from birth to adulthood. *Neuroimage*. 2009;47. doi:10.1016/s1053-8119(09)70884-5

8. Wang S, Peterson DJ, Gatenby JC, Li W, Grabowski TJ, Madhyastha TM. Evaluation of field map and nonlinear registration methods for correction of susceptibility artifacts in diffusion MRI. *Front Neuroinform*. 2017;11. doi:10.3389/fninf.2017.00017

9. Huntenburg JM, Gorgolewski KJ, Anwander A, Margulies DS. Evaluating nonlinear coregistration of BOLD EPI and T1 images. In: *20th Annual Meeting of the Organization for Human Brain Mapping*. ; 2014.

10. Treiber JM, White NS, Steed TC, et al. Characterization and correction of geometric distortions in 814 Diffusion Weighted Images. *PLoS One*. 2016;11(3). doi:10.1371/journal.pone.0152472

11. Greve DN, Fischl B. Accurate and robust brain image alignment using boundary-based registration. *Neuroimage*. 2009;48(1). doi:10.1016/j.neuroimage.2009.06.060

12. Jenkinson M, Bannister P, Brady M, Smith S. Improved optimization for the robust and accurate linear registration and motion correction of brain images. *Neuroimage*. 2002;17(2). doi:10.1016/S1053-8119(02)91132-8

13. Glasser MF, Sotiropoulos SN, Wilson JA, et al. The minimal preprocessing pipelines for the Human Connectome Project. *Neuroimage*. 2013;80. doi:10.1016/j.neuroimage.2013.04.127

14. Pruim RHR, Mennes M, van Rooij D, Llera A, Buitelaar JK, Beckmann CF. ICA-AROMA: A robust ICA-based strategy for removing motion artifacts from fMRI data. *Neuroimage*. 2015;112. doi:10.1016/j.neuroimage.2015.02.064

15. Power JD, Mitra A, Laumann TO, Snyder AZ, Schlaggar BL, Petersen SE. Methods to detect, characterize, and remove motion artifact in resting state fMRI. *Neuroimage*. 2014;84. doi:10.1016/j.neuroimage.2013.08.048

16. Behzadi Y, Restom K, Liau J, Liu TT. A component based noise correction method (CompCor) for BOLD and perfusion based fMRI. *Neuroimage*. 2007;37(1). doi:10.1016/j.neuroimage.2007.04.042

17. Satterthwaite TD, Elliott MA, Gerraty RT, et al. An improved framework for confound regression and filtering for control of motion artifact in the preprocessing of resting-state functional connectivity data. *Neuroimage*. 2013;64(1). doi:10.1016/j.neuroimage.2012.08.052

18. Lanczos C. Evaluation of Noisy Data. *Journal of the Society for Industrial and Applied Mathematics Series B Numerical Analysis*. 1964;1(1). doi:10.1137/0701007

19. Abraham A, Pedregosa F, Eickenberg M, et al. Machine learning for neuroimaging with scikit-learn. *Front Neuroinform*. 2014;8(FEB). doi:10.3389/fninf.2014.00014

Table S1. Mentalizing Region Peak Voxels and AAL Atlas Labels

| Cluster Name | Peak Coordinate [x,y,z] | Volume (# Voxels) | AAL Cluster Labelling |  |
| --- | --- | --- | --- | --- |
| R_TPJ | [56, -50, 26] | 1480 | 29.78% Angular_R;  24.81% Temporal_Mid_R; 17.92% SupraMarginal_R; 15.43% Temporal_Sup_R;  6.44% no_label |  |
| Precuneus | [2, -58, 38] | 1930 | 44.40% Precuneus_L  35.25% Precuneus_R |  |
| R_ STS | [52, -8, -14] | 1229 | 40.05% Temporal_Mid_R; 26.67% Temporal_Sup_R; 21.50% Temporal_Pole_Mid_R;  9.95% Temporal_Pole_Sup_R |  |
| L_Angular Gyrus | [-58, -64, 24] | 1125 | 36.77% Angular_L;  32.31% no_label;  23.70% Temporal_Mid_L | |
| L_MTG | [-54, 6, -24] | 51 | 78.18% Temporal_Mid_L; 16.36% Temporal_Pole_Sup_L; 5.45% Temporal_Pole_Mid_L | |
| L_Cerebellum | [-24, -84, -34] | 53 | 57.38% Cerebellum_Crus2_L; 42.62% Cerebellum_Crus1_L | |
| L_STS | [-58, -14, -10] | 122 | 100.00% Temporal_Mid_L | |
| mPFC | [4, 66, -10] | 14 | 95.45% Frontal_Med_Orb_R | |
| dmPFC | [6, 54, 38] | 147 | 65.64% Frontal_Sup_Medial_R; 33.85% Frontal_Sup_Medial_L | |
| L_SMA | [-26, 30, 44] | 40 | 53.33% Frontal_Sup_2_L; 46.67% Frontal_Mid_2_L | |
| L_MTG2 | [-50, -30, -6] | 28 | 75.68% Temporal_Mid_L; 24.32% no_label | |
| R_dlPFC | [26, 26, 40] | 59 | 55.00% Frontal_Mid_2_R; 45.00% Frontal_Sup_2_R | |

Note. When present, R = right and L = left. TPJ = temporoparietal junction. STS = superior temporal sulcus. MTG = middle temporal gyrus. mPFC = medial prefrontal cortex. dmPFC = dorsomedial prefrontal cortex. SMA = supplementary motor area. dlPFC = dorsolateral prefrontal cortex.

**Table S2.** Associations of Regional Activation with Group (controlling for age and sex)

| Brain Region | Group  (Patient = 1, Control = 0) | | |
| --- | --- | --- | --- |
| R_TPJ  [56, -50, 26] | -1.224  (p = .216) | | |
|  |  | | |
| Precuneus  [2, -58, 38] | -0.939  (p = .410) | | |
|  |  | | |
| R_STS  [52, -8, -14] | 1.201  (p = .297) | | |
|  |  | | |
| L_AngularGyrus  [-58, -64, 24] | 0.391  (p = .741) | | |
|  |  | | |
| L_MTG1  [-54, 6, -24] | 2.065*  (p = .029) | | |
|  |  | | |
| L_Cerebellum  [-24, -84, -34] | 2.253*  (p = .026) | | |
|  |  | | |
| L_STS  [-58, -14, -10] | -2.803*  (p = .026) | | |
|  |  | | |
| mPFC  [4, 66, -10] | 0.623  (p = .511) | | |
|  |  | | |
| dmPFC  [6, 54, 38] | -0.774  (p = .477) | | |
|  |  | | |
| L_SMA  [-26, 30, 44] | 1.624  (p = .163) | | |
|  |  | | |
| L_MTG2  [-50, -30, -6] | 0.373  (p = .655) | | |
|  |  | | |
| R_dlPFC  [26, 26, 40] | -2.372*  (p = .023) | | |
|  |  |  |  |

*Note.* *p<.05.The group column represents the results of a logistic regression model, using brain activation estimates from the false belief task to predict group status (i.e., participants with psychiatric diagnoses versus non-clinical controls). Standardized regression coefficients are presented. When present, R = right and L = left. TPJ = temporoparietal junction. STS = superior temporal sulcus. MTG = middle temporal gyrus. mPFC = medial prefrontal cortex. dmPFC = dorsomedial prefrontal cortex. SMA = supplementary motor area. dlPFC = dorsolateral prefrontal cortex.

**Table S3.** Hierarchical Linear Regression of Regional Activation with Social Functioning (controlling for age and sex)

| Model | | Model Statistics | | | | | | Predictor Statistics | |  |
| --- | --- | --- | --- | --- | --- | --- | --- | --- | --- | --- |
|  |  | R^2^(ΔR^2^) | | F | | *p* | | β | *p* |  |
|  |  |  |  |  |  |  |  |  |  |  |
| *Model 1* | | 0.212 (0.045) | | 1.336 | | 0.271 | |  |  |  |
| Age | |  | |  | |  | | -0.012 | 0.929 |  |
| Sex | |  | |  | |  | | 0.211 | 0.110 |  |
| *Model 2* | | 0.210 (0.165) | | 0.873 | | 0.572 | |  |  |  |
| Age | |  | |  | |  | | -0.154 | 0.311 |  |
| Sex | |  | |  | |  | | 0.172 | 0.247 |  |
| R_TPJ | |  | |  | |  | | 0.025 | 0.896 |  |
| Precuneus | |  | |  | |  | | 0.068 | 0.762 |  |
| R_STS | |  | |  | |  | | -0.091 | 0.693 |  |
| L_Angular Gyrus | |  | |  | |  | | 0.042 | 0.867 |  |
| L_MTG | |  | |  | |  | | -0.209 | 0.266 |  |
| L_STS | |  | |  | |  | | 0.432 | 0.058 |  |
| mPFC | |  | |  | |  | | -0.188 | 0.343 |  |
| dmPFC | |  | |  | |  | | -0.082 | 0.716 |  |
| L_SMA | |  | |  | |  | | -0.162 | 0.457 |  |
| L_MTG2 | |  | |  | |  | | -0.132 | 0.453 |  |
| R_dlPFC | |  | |  | |  | | 0.266 | 0.162 |  |
| *Model 3* | | 0.270 (0.061) | | 3.732 | | 0.060 | |  |  |  |
| Age | |  | |  | |  | | -0.140 | 0.345 |  |
| Sex | |  | | |  |  | | 0.162 | 0.261 |  |
| R_TPJ | |  | | |  |  | | 0.088 | 0.641 |  |
| Precuneus | |  | | |  |  | | 0.205 | 0.373 |  |
| R_STS | |  | | |  |  | | -0.153 | 0.498 |  |
| L_Angular Gyrus | |  | | |  |  | | 0.009 | 0.971 |  |
| L_MTG | |  | | |  |  | | -0.288 | 0.126 |  |
| L_STS | |  | | |  |  | | 0.568 | 0.018 |  |
| mPFC | |  |  | | | |  | -0.180 | 0.350 |  |
| dmPFC | |  |  | | | |  | 0.062 | 0.789 |  |
| L_SMA | |  |  | | | |  | -0.333 | 0.150 |  |
| L_MTG2 | |  |  | | | |  | -0.129 | 0.453 |  |
| R_dlPFC | |  |  | | | |  | 0.370 | 0.057 |  |
| L_Cerebellum | |  |  | | | |  | -0.379 | 0.060 |  |

Note. When present, R = right and L = left. TPJ = temporoparietal junction. STS = superior temporal sulcus. MTG = middle temporal gyrus. mPFC = medial prefrontal cortex. dmPFC = dorsomedial prefrontal cortex. SMA = supplementary motor area. dlPFC = dorsolateral prefrontal cortex.
